# Next-generation atrial fibrillation ablation: clinical performance of pulsed-field ablation and very high-power short-duration radiofrequency

**DOI:** 10.1007/s10840-024-01853-4

**Published:** 2024-07-13

**Authors:** Nibras Soubh, Judith Gronwald, Helge Haarmann, Eva Rasenack, Philipp Bengel, Simon Schlögl, Gerd Hasenfuß, Markus Zabel, Leonard Bergau

**Affiliations:** 1 University Medical Center Göttingen (UMG), Robert-Koch-Str. 40, 37075 Göttingen, Germany; 2 Department of Cardiology, Justus-Liebig University of Giessen, Giessen, Germany

**Keywords:** Pulmonary vein isolation, Electroporation, High-power short duration, Single-shot ablation, Comparison

## Abstract

**Introduction:**

Pulsed-field energy (PFA) and very high-power short-duration radiofrequency (vHPSD-RF) are two novel ablation methods for pulmonary vein isolation (PVI). Both PFA and vHPSD-RF show promise for improving efficacy, safety, and reducing procedure durations. However, direct comparisons between these two techniques are scarce.

**Methods and results:**

Retrospective analysis of 82 patients with symptomatic AF. Of these, 52 patients received PFA and 30 received vHPSD-RF (90 W, 4 s) as index procedure. At the 6-month follow-up, AF recurrence occurred in 4 patients following PFA and 5 patients following vHPSD-RF (*p*-value = 0.138). Significant improvements in the EHRA and NYHA stages were evident in both PFA (*p* < 0.001 and *p* = 0.047, respectively) and vHPSD-RF groups (*p* = 0.007 and *p* = 0.012, respectively). The total procedure duration and the left atrial dwell time were significantly shorter in the PFA group (64 ± 19 min vs. 99 ± 32 min, *p* < 0.001 and 41 ± 12 min vs. 62 ± 29 min, *p* < 0.001, respectively). The fluoroscopy time and dose area product were significantly higher in PFA (14 ± 6 vs. 9 ± 5 min, *p* < 0.001 and 14 ± 9 vs. 11 ± 9 Gy cm^2^, *p* = 0.046, respectively). One patient in the vHPSD-RF group suffered a stroke, not directly linked to the procedure (0 vs. 1 major complication, *p* = 0.366).

**Conclusion:**

Based on this retrospective single-center study, PFA and vHPSD-RF were associated with similar effectiveness and safety profiles. PFA was linked to shorter procedure times and higher radiation exposure compared to vHPSD-RF.

**Graphical Abstract:**

**Figure:**
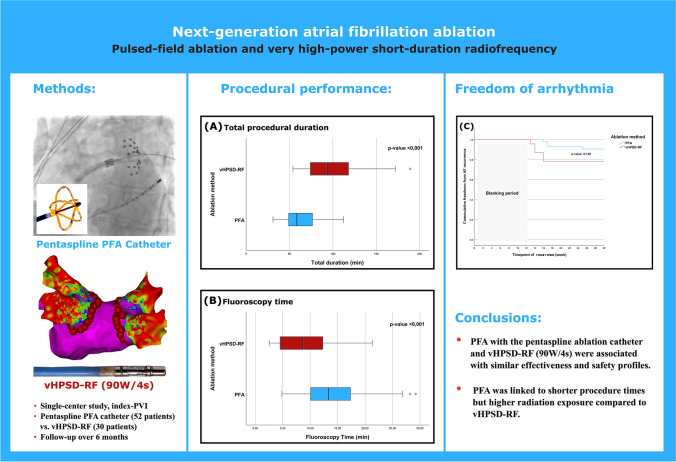
Summarizing the study methods and results. PFA has been found to be associated with shorter procedure durations (**A**) and longer fluoroscopy times (**B**). No significant difference has been detected regarding the recurrence of atrial fibrillation in the first 6 months (**C**). Abbreviations: PFA, pulsed-field ablation; vHPSD-RF, very high-power short-duration radiofrequency; PVI, pulmonary vein isolation; W, watts

## Introduction

Pulmonary vein isolation (PVI) is recommended in patients with drug refractory symptomatic atrial fibrillation (AF) [1]. Various ablation methods have been developed and refined to optimize the procedure’s precision and outcomes. These methods encompass a spectrum of thermal techniques, including radiofrequency (RF), cryoballoon ablation (CRA), and laser ablation, as well as a non-thermal ablation technique, namely pulsed-field ablation (PFA). Radiofrequency catheter ablation employs point-by-point applications of RF energy to create localized thermal lesions within the myocardial tissue. Myocardium exposed to high temperatures undergoes irreversible coagulative necrosis, evolving into non-conducting myocardial scar [2]. Besides other factors, the lesion size and depth are mainly determined by the catheter contact force, delivered power, and ablation duration [3]. The next-generation RF catheters, for example, the QDOT MICRO™ catheter (Biosense Webster, CA, USA), deliver single applications up to 90 W for 4 s. This very high-power short-duration radiofrequency ablation (vHPSD-RF) protocol has been shown to increase the lesion volumes and width/depth ratios [4]. Consequently, the resulting ablation lesions tend to be relatively shallower, potentially providing a protective effect on neighboring organs [5]. Unlike RF, laser ablation, and cryoballoon ablation, in the pulsed-field ablation (PFA), the cellular membrane of the cardiomyocytes is non-thermally destabilized by application of high-voltage ultra-rapid electrical pulses. This results in nanoscale pores of the cell membrane (electroporation) with consecutive leakage of cell contents leading to cell death. The energy threshold to create permanent lesions varies depending on the tissue type, with cardiomyocytes being more sensitive to pulsed-field energy compared to other tissue types. Therefore, permanent ablation of cardiomyocyte tissue can be achieved while the extracellular matrix and adjacent tissues are spared [6]. This tissue-selective approach appears to be a unique advantage of PFA. Both PFA and vHPSD-RF are effective and safe in patients with AF. Moreover, both ablation methods harbor a potential for preventing “collateral damage” and shortening the procedure times [7–10]. To date, no direct comparison between PFA and vHPSD-RF using the QDOT MICRO™ catheter has been conducted.

In this single-center study, we performed a head-to-head comparison of these two next-generation ablation methods with a focus on procedural performance and safety.

## Methods

### Study design

We retrospectively analyzed the medical records of all patients who underwent their index pulmonary vein isolation (PVI) in our center from May 1, 2022, to June 30, 2023. All patients who underwent PVI using PFA or vHPSD-RF were eligible for the inclusion. The primary endpoint was procedure efficacy surrogated by the arrhythmia-free survival during the follow-up. Secondary endpoints encompassed procedural success, defined as isolation of all pulmonary veins at the end of the procedure, improvement of AF and heart failure symptoms, procedure duration, left atrium (LA) dwell time, fluoroscopy time, dose area product (DAP), volume of used contrast media, length of hospital stay, and major complications. Major complications were defined as death, stroke or transient ischemic attack (TIA), cardiac tamponade, atrial-esophageal fistula, and vascular access complications requiring surgical interventions. A 90-day post-ablation period (blanking period) was instituted, during which no formal follow-ups were conducted. Patients were, however, strongly encouraged to promptly notify us of any recurrent symptoms. In case of AF recurrence in the blanking period, an early restoration of sinus rhythm was targeted. All patients were scheduled for follow-up visits in our outpatient clinic 6 months after the ablation. Those visits included medical history, clinical assessment, ECG Holter monitoring for at least 3 days, and a transthoracic echocardiography. The study was approved by the local ethics committee (Approval No:11/07/23) and conducted in accordance with the declaration of Helsinki.

### Procedural preparations and intraprocedural management

Intracardiac thrombi were excluded using transesophageal echocardiography (TEE) in patients with insufficient oral anticoagulation or very high thromboembolic risk, defined as a previous intracardiac thrombus or thromboembolic stroke. Other patients with sufficient uninterrupted oral anticoagulation were not required to undergo a TEE before the ablation. The oral anticoagulation paused only on the morning of the procedure and resumed on the same day in patients with no signs of active bleeding (maximal interruption of 12 h). All ablation procedures were performed under deep sedation using propofol with continuous monitoring of the peripheral oxygen saturation and end-tidal carbon dioxide (etCO2) via nasal cannula capnography along with non-invasive blood pressure measurements. 12-lead ECG and intracardiac signals were recorded using CardioLab 7.0 (GE Medical Systems, USA). In all procedures, two sheaths (7 French) were inserted into the left femoral vein, and one sheath (8 French) was inserted into the right femoral vein. A diagnostic decapolar catheter (Inquiry, Abbott, USA) was positioned in the coronary sinus. The transseptal puncture (TSP) with the Brockenbrough needle (BRK-1, Abbott, USA) was performed under fluoroscopic and pressure guidance using a non-steerable sheath (Fast-Cath SL1, 8,5 French, Abbott, USA) in patients scheduled for PFA ablation and a steerable sheath (CARTO Vizigo, Biosense Webster, USA) in patients planned for vHPSD-RF. During the procedure, an activated clotting time (ACT) of > 300 s was maintained. Fluoroscopy with two pulses per second was used for catheters placement and TSP. In all procedures, contrast agent was applied for selective pulmonary vein angiography. At the end of procedure, pericardial effusion (PE) was ruled out using echocardiography and the skin puncture sites were closed using figure-of-8 sutures. All patients were observed over the night in the cardiology ward and evaluated for possible discharge on the following day.

### Pulsed-field ablation

**Fig. 1:**
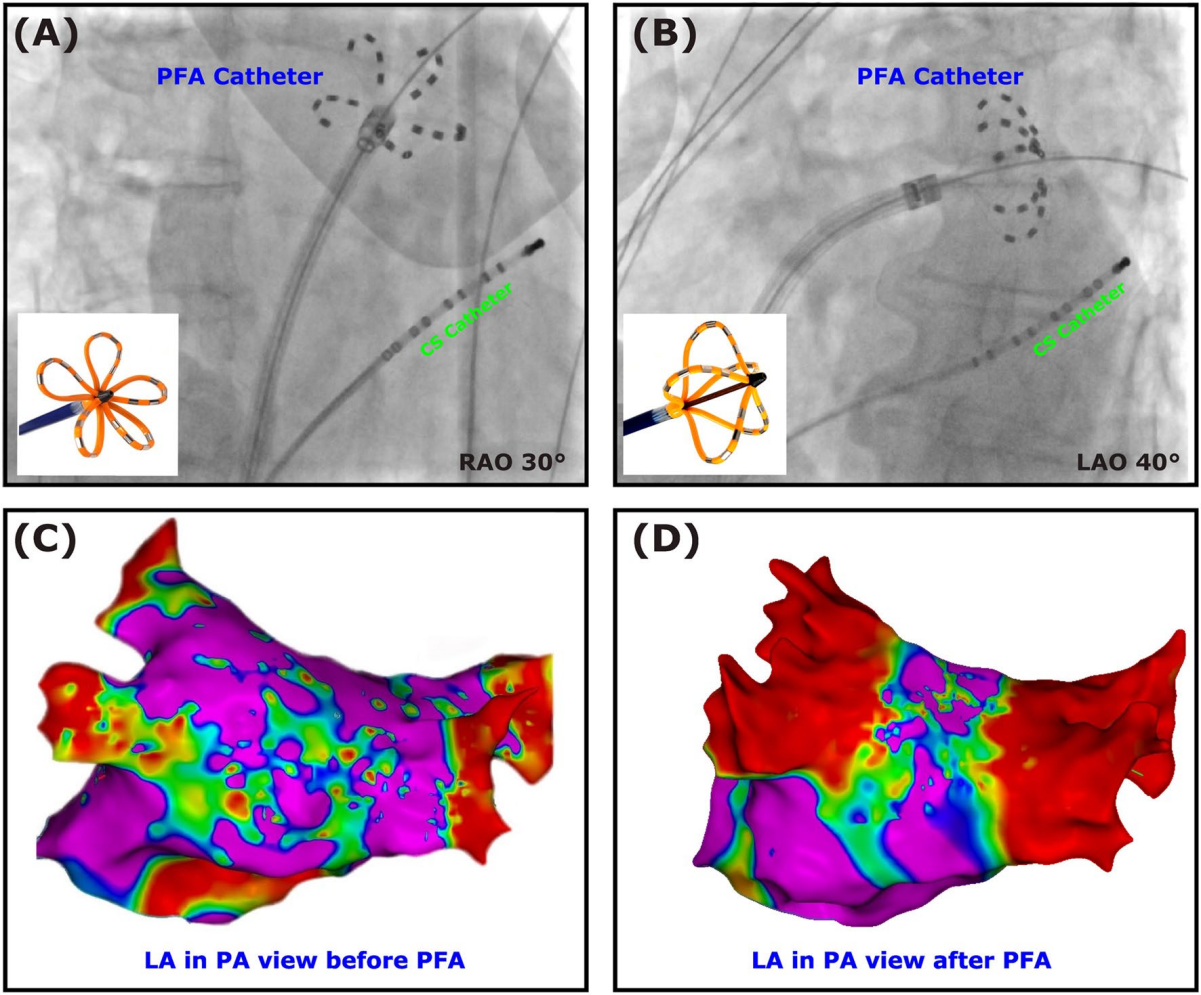
Fluoroscopic image and corresponding electroanatomic Map during pulsed-field ablation (PFA). The coronary sinus (CS) catheter in the CS. The pulsed-field ablation (PFA) catheter (“flower” configuration) in the right superior pulmonary vein in the right anterior oblique view 30° (**A**) and in the “basket” configuration in the left inferior pulmonary vein in the left anterior oblique view 40° (**B**). Electroanatomical map of the left atrium (LA) in the posterior-anterior (PA) view (CARTO® 3 System, Biosense Webster, USA) before PFA (**C**) and after PFA (**D**)

After selective pulmonary vein angiography, an anatomical and voltage mapping in the left atrium was performed using a 3D mapping system (Ensite X, Abbott, USA, or CARTO® 3 System, Biosense Webster, USA) and a mapping catheter, the Advisor™ HD Grid catheter (Abbott, USA) or the Pentaray™ NAV ECO (Biosense Webster, USA). After that, the SL1 sheath was replaced by the steerable PFA sheath (Faradrive, Boston Scientific, USA). According to the manufacturer’s protocol, pulsed-field energy was applied 8 times per pulmonary vein at ostial positions, 4 times in “basket” configuration and 4 times in “flower” configuration (Fig. 1). At the end of procedure, a LA re-map was performed to visualize the ablation lesions and ensure complete isolation of all veins.

### Very high-power short-duration radiofrequency ablation

**Fig. 2:**
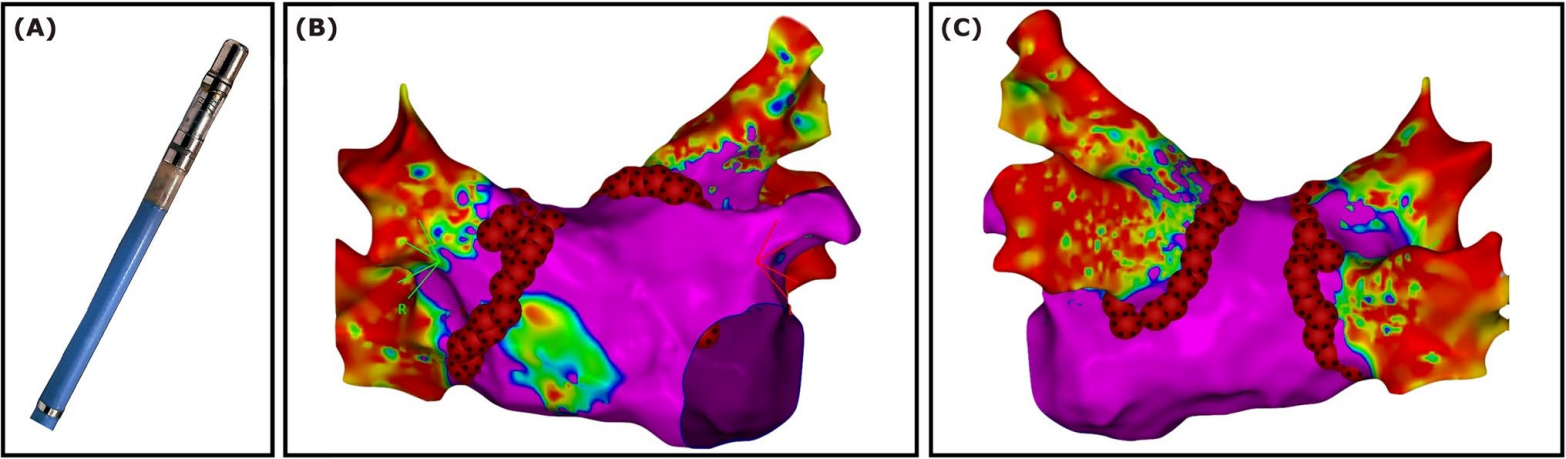
Very high-power short-duration radiofrequency ablation (vHPSD-RF) using the QDOT MICRO™ catheter (Biosense Webster, CA, USA) (**A**). Electroanatomic voltage maps (0.05–0.5 mV) of the left atrium in anterior-posterior view (**B**) and posterior-anterior view (**C**) with the vHPSD ablation tags

After selective pulmonary vein angiography, a high-density anatomical and voltage mapping in the left atrium was performed using the PENTARAY® NAV ECO mapping catheter and the CARTO® 3 System (Biosense Webster, USA). After the pulmonary vein ostia were marked, circumferential lesions were created employing the QDOT MICRO™ catheter (Biosense Webster, USA) and the QMode + (90 watts for 4 s, targeted interlesion distance 4 mm) (Fig. 2). Further ablation lines were applied if needed.

### Statistical analysis

The normality of the acquired data was assessed using the Shapiro–Wilk test. A significance level of 0.05 was employed for all statistical tests, implying that results with a *p*-value less than 0.05 were considered statistically significant. Between-group comparisons were performed using independent samples *t*-tests for normally distributed data and the Mann–Whitney *U*-test for non-normally distributed data. For nominal data, Fisher’s exact test was employed. The log-rank test was used to evaluate AF recurrences between the two groups. Additionally, a multivariate Cox proportional hazards regression model was applied to account for potential confounders. Time effects were assessed using Wilcoxon signed-rank test. The statistical analysis was performed using SPSS (V.29.0.0.0/241). Figures were generated using SPSS and Inkscape (version 1.2).

## Results

### Patient’s characteristics

A total of 396 patients who received pulmonary vein isolation at our center from May 1, 2022, to June 30, 2023, were screened. Of these, 52 patients received their index PVI using PFA and 30 patients using the very high-power short-duration radiofrequency (vHPSD-RF) protocol (Q Mode + , 90 W, 4 s). Patients in the vHPSD-RF group were significantly older (67 ± 10 vs. 73 ± 7, *p* = 0,009), with a higher prevalence of coronary artery disease (17% vs 43%, *p* = 0.019) and higher left atrial volume index (LAVI) (39 ± 14 vs. 48 ± 18 ml/m^2^, *p* = 0.025). All baseline characteristics are summarized in Table 1.

**Table 1:** Baseline characteristics

	PFA	vHPSD-RF	*p*-value
Patients’ characteristics			
*N*	**52**	**30**	
Age (mean ± SD, years)	67 ± 10	73 ± 7	**0.009**
AF clinical type			0.43
- Paroxysmal AF (%)	25 (48)	13 (43)	
- Persistent AF (%)	27 (52)	17 (57)	
Gender			**0.025**
- Female (%)	15 (29)	16 (53)	
- Male (%)	37 (71)	14 (47)	
BMI (mean ± SD, kg/m^2^)	28 ± 6	27 ± 6	0.84
Medical history			
- CHF (%)	20 (38)	13 (43)	0.42
- CAD (%)	9 (17)	13 (43)	**0.019**
- HTN (%)	43 (83)	25 (83)	0.6
- DM (%)	8 (15)	4 (13)	0.54
- HLP (%)	25 (48)	11 (37)	0.22
- Smoking history (%)	11 (21)	3 (10)	0.16
- Stroke or TIA (%)	5 (10)	4 (13)	0.43
- OSAS (%)	8 (15)	5 (17)	0.56
- COPD (%)	5 (10)	4 (13)	0.43
- LEAD (%)	3 (6)	1 (3)	0.53
- CHA_2_DS_2_-VASc-Score (mean ± SD)	4 ± 1.6	4 ± 1.7	0.56
Echocardiographic findings			
- LVEF (mean ± SD, %)	52 ± 8	47 ± 14	0.96
- LAVI (mean ± SD, ml/m^2^)	39 ± 14	48 ± 18	**0.025**
- AS (%)	3 (6)	2 (7)	1
- MR (%)	17 (33)	15 (50)	0.16
- TR (%)	13 (25)	12 (40)	0.213
- AR (%)	5 (10)	5 (17)	0.485
Medication			
- Betablocker (%)	37 (71)	21 (70)	1
- Antiarrhythmic drugs (%)	14 (27)	5 (17)	0.416
- Digitalis (%)	1 (2)	0 (0)	1
NT-ProBNP level (ng/l)	1238 ± 3000	2360 ± 3404	0.08

*PFA* pulsed-field ablation, *vHPSD-RF* very high-power short-duration radiofrequency, *SD* standard deviation, *AF* atrial fibrillation, *CHF* congestive heart failure, *CAD* coronary artery disease, *HTN* arterial hypertension, *DM* diabetes mellitus, *HLP* hyperlipoproteinemia, *TIA* transient ischemic attack, *OSAS* obstructive sleep apnea syndrome, *COPD* chronic obstructive pulmonary disease, *LEAD* lower extremity atherosclerotic disease, *LVEF* left ventricular ejection fraction, *LAVI* left atrial volume index, *AS* aortic stenosis, *MR* mitral valve regurgitation, *TR* tricuspid valve regurgitation, *AR* aortic valve regurgitation

### Clinical outcome

**Fig. 3:**
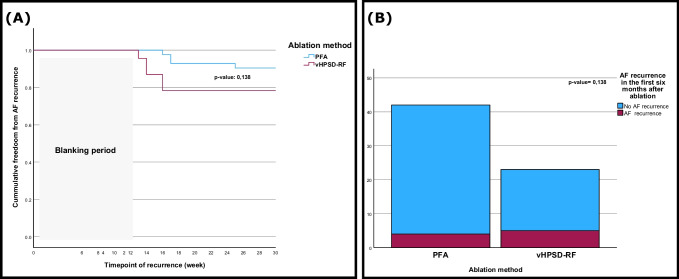
Kaplan-Meier curve (**A**) demonstrating atrial fibrillation (AF) recurrences after a blanking period of 3 months in the pulsed-field ablation (PFA) and very high-power short-duration radiofrequency ablation (vHPSD-RF) groups. Bar graph (**B**) shows a direct comparison of the recurrence rate in the PFA and vHPSD-RF group. No statistically significant difference was detected (*p* = 0.138, log-rank test)

In the blanking period, 7 patients (13%) in the PFA group and 5 patients (17%) in the vHPSD-RF group had symptomatic AF episodes (*p* = 0.741). Most patients appeared for their scheduled follow-up visits (83% vs. 77% in the PFA and vHPSD-RF respectively, *p* = 0.569). In both groups, patients reported an improvement of their AF-related symptoms with a significant lowering of their European Heart Association (EHRA) stage (*p* < 0.001 and *p* = 0.007 in the PFA and vHPSD-RF groups respectively). Similarly, the New York Heart Association (NYHA) stage has significantly improved after ablation in both groups (*p* = 0.047 and 0.012 in the PFA and vHPSD-RF groups respectively) (Table 2). At the 6-month mark, no significant differences regarding AF recurrence were observed (4 patients (9%) in the PFA group and 5 patients (17%) in the vHPSD-RF, *p* = 0.138) (Table 3). Figure 3 illustrates the AF recurrence within the first 6 months following ablation. The multivariate Cox proportional hazards regression analysis revealed that age (HR = 1.056, 95% CI [0.930–1.198], *p* = 401), LAVI (HR = 1.062, 95% CI [0.991–1.137], *p* = 0.087), and presence of CAD (HR = 0.115, 95% CI [0.002–5.855], *p* = 0.281) were not statistically significant predictors of atrial fibrillation recurrence in our cohort.

**Table 2:** Effects of pulmonary vein ablation on clinical symptoms assessed using EHRA and NYHA scores before and after ablation

	Before ablation Median (1st quartile–3rd quartile)	After ablation Median (1st quartile–3rd quartile)	*p*-value
PFA			
EHRA stage	2 (2–3)	1 (1–2)	*p* < 0.001
NYHA stage	1 (1–3)	1 (1–2)	*p* = 0.047
vHPSD-RF			
EHRA stage	2.5 (1–3)	2 (1–2)	*p* = 0.007
NYHA stage	2 (2–3)	2 (1–2)	*p* = 0.012

*PFA* pulsed-field ablation, *EHRA* European Heart Association, *NYHA* New York Heart Association, *vHPSD-RF* very high-power short-duration radiofrequency

**Table 3:** Procedural success and recurrence of atrial fibrillation in the blanking period and in the first 6 months following PVI using PFA and vHPSD-RF

	PFA	vHPSD-RF	*p*-value
*N*	**52**	**30**	
Clinical outcome			
Primary success (%)	52 (100)	30 (100)	1
AF in the blanking period (%)	7 (13)	5 (17)	0.741
6-month interval			
- *N* of follow-up visits (%)	43 (83)	23(77)	0.569
- Recurrences (%)	4 (9)	5 (22)	0.138

*PFA* pulsed-field ablation, *vHPSD-RF* very high-power short-duration radiofrequency, *AF* atrial fibrillation

### Procedural parameters

**Fig. 4:**
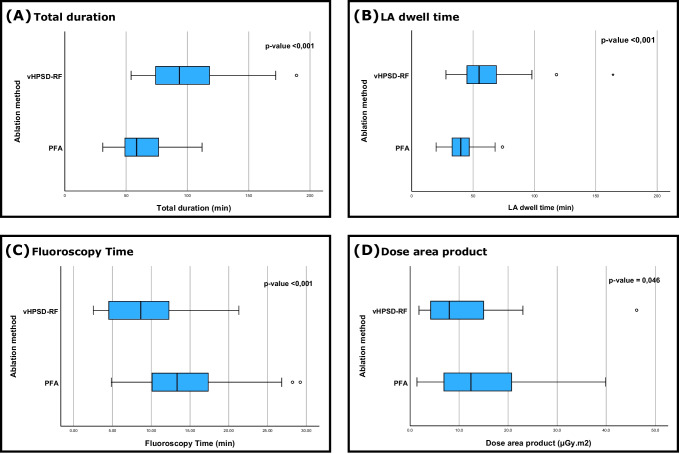
Comparison of total duration (**A**), LA dwell time (**B**), fluoroscopy time (**C**), and dose area product DAP (**D**) between pulsed-field ablation (PFA) and very high-power short-duration radiofrequency ablation (vHPSD-RF). PFA was associated with significantly shorter total duration and LA times (*p*-values < 0.001, Mann–Whitney *U*-test), while vHPSD-RF had lower radiation exposure with a significantly shorter fluoroscopy time and DAP (*p*-values < 0.001 and 0.046, respectively, Mann–Whitney *U*-test)

Total procedural duration (skin to skin time) and left atrial (LA) dwell time were significantly shorter in PFA compared to vHPSD-RF (64 ± 19 vs. 99 ± 32 min and 41 ± 12 vs. 62 ± 29 min respectively, *p*-values < 0.001). In contrast, fluoroscopy time and dose area product (DAP) were significantly higher in PFA (14 ± 6 vs. 9 ± 5 min, *p* = 0.001, and 14 ± 9 vs. 11 ± 9 Gy cm^2^, *p* = 0.046, respectively) (Fig. 4). In a sub-analysis where patients with additional right atrial ablations were excluded (only left atrial ablations), PFA was still associated with shorter total procedure time (63 ± 18 vs. 92 ± 34 min, *p* < 0.001) and LA dwell time (41 ± 12 vs. 60 ± 34 min, *p* = 0.03). The duration of hospitalization and contrast media volume showed no statistically significant differences. At the end of the procedures, all PVs were isolated, 13 patients (43%) in the vHPSD-RF group had concomitant typical atrial flutter and therefore underwent a cavotricuspid isthmus (CTI) ablation, while only one patient (2%) in the PFA group had a conventional RF CTI ablation in addition to PVI (*p* < 0.001). In 9 patients (30%) in the vHPSD-RF group, further ablation lines in LA were applied (*p* < 0.001). No statistically significant differences were observed regarding major complications. While no major complications occurred in the PFA group, one patient in the vHPSD-RF group suffered an embolic stroke 2 days after the procedure linked to an interruption of the oral anticoagulation against our recommendation. All procedural parameters are summarized in Table 4.

**Table 4:** Procedural parameters of PFA and vHPSD-RF and major complications, only one complication was detected (stroke due to interruption of anticoagulation against our recommendations)

	PFA	vHPSD-RF	*p*-value
*N*	**52**	**30**	
Procedural parameters			
Total duration (min)	64 ± 19	99 ± 32	** < 0.001**
LA dwell time (min)	41 ± 12	62 ± 29	** < 0.001**
Fluoroscopy time (min)	14 ± 6	9 ± 5	** < 0.001**
DAP (Gy cm^2^)	14 ± 9	11 ± 9	**0.046**
Contrast media (ml)	11 ± 4	12 ± 2	0.134
Hospital stay (days)	3 ± 2.8	5 ± 5.5	0.58
Ablation targets			
- PVI	52 (100)	30 (100)	1
- PVI + CTI ablation	1 (2)	13 (43)	** < 0.001**
- PVI + further targets in LA	0 (0)	9 (30)	** < 0.001**
Major complications	0 (0)	1 (3)	0.366
- Death	0 (0)	0 (0)	1
- Stroke/TIA	0 (0)	1 (3)	0.366
- Cardiac tamponade	0 (0)	0 (0)	1
- Atrial-esophageal fistula	0 (0)	0 (0)	1
- Vascular access complications	0 (0)	0 (0)	1

*PFA* pulsed-field ablation, *vHPSD-RF* very high-power short-duration radiofrequency, *LA* left atrium, *DAP* dose area product, *PVI* pulmonary veins isolation, *CTI* cavotricuspid isthmus, *TIA* transient ischemic attack, *AF* atrial fibrillation

## Discussion

### Main findings

In the present study, we compared the clinical performance of two novel ablations systems, pulsed-field ablation, and very high-power short-duration radiofrequency ablation. We observed the following findings:

1. Both ablation systems showed comparable short- and mid-term safety and efficacy profiles.
2. Pulsed-field ablation was associated with shorter procedure times.
3. The very high-power short-duration approach was associated with shorter radiation exposure and might be favorable for patients with concomitant arrhythmias requiring linear ablation.

### Procedural characteristics

Since the rediscovery of pulsed-field ablation in 2019 [11], the clinical evidence regarding the safety and efficacy of this novel ablation method has been accumulating [8]. However, there are only few clinical trials that performed a direct comparison of PFA with established thermal ablation methods. Clinical trials have shown that PFA is associated with a shorter procedure duration compared to CRA [12], while others found PFA and CRA to have similar procedural characteristics [13].

The new QDOT micro-ablation catheter (Biosense Webster, CA, USA) has been designed to deliver high-power radiofrequency energy (up to 90 W) during a short period of time (up to 4 s), enabling rapid point-by-point ablation procedures [14]. The QDOT micro-catheter was shown to be superior to conventional “low-power” ablation index (AI)-based ablation protocols regarding procedure and ablation durations [9] [10]. Our study is the first head-to-head comparison of PFA and vHPSD-RF using QDOT micro-catheter.

At baseline, patients in the vHPSD-RF group were significantly older with higher left atrial volumes, which can be attributed to our ablation method selection strategy. In our center, single-shot ablation methods (CRA and PFA) are preferred for index PVI in patients with no other concomitant arrhythmias and where no modifiable substrate is expected. On the other hand, point-by-point methods (conventional and next-generation RF) are preferred in patients with expected modifiable substrate, documented, or suspected other atrial arrhythmias or previous atrial ablations. This group of patients typically has a higher risk of left atrial enlargement [15]. Both vHPSD-RF and PFA were previously shown to be associated with remarkably short procedure times [9, 16]. Although we combined all our PFA procedures with electroanatomical mapping, the total procedure time was still significantly shorter than in vHPSD-RF. Similar results were observed in a recently published retrospective clinical study that compared PFA with another vHPSD-RF ablation catheter (flexibility, Abbott, USA) using 70 W for 7 s [17]. While only one patient in the PFA group underwent a CTI ablation due to spontaneous onset of typical atrial flutter during PFA, 13 patients in the vHPSD-RF group underwent CTI ablations due to concomitant typical atrial flutter. This might have prolonged the total duration of these procedures. However, even when those patients were excluded, PFA was still associated with shorter total procedure time (63 ± 18 vs. 92 ± 34 min, *p* < 0.001) and LA dwell time (41 ± 12 vs. 60 ± 34 min, *p* = 0.03). As reported in previous clinical trials that compared PFA to conventional radiofrequency ablations [16], our study showed that PFA was associated with significantly higher radiation exposure when compared to vHPSD-RF. This is probably the result of the fluoroscopy-guided catheter navigation in PFA.

### Procedural success

The efficacy and safety of PFA were shown to be comparable to cryoballoon ablation (CRA) [12, 13]. The recently published ADVENT trial [18] proved PFA to be noninferior to thermal ablation methods (RF and CRA) regarding efficacy and safety at 1 year. A recently published meta-analysis, that included the ADVENT trial and five other prospective clinical trials, found that PFA was associated with shorter procedure durations and longer fluoroscopy times when compared to thermal ablation methods [16]. Yet, in all previously mentioned trials, PFA was compared either to CRA or to conventional RF ablation catheters (RF energy ≤ 50 W). In this respect, our study adds a new important value as the novel vHPSD-RF approach is now compared to PFA. Our results are in accordance with the aforementioned trials regarding the success rate at 6 months. The survival analysis was adjusted by age, LAVI, and presence of CAD, as these variables exhibited statistically significant differences between the PFA and vHPSD-RF groups at baseline, indicating potential confounding effects. However, the multivariate Cox proportional hazards model did not identify a significant influence of these covariates on the risk of AF recurrence in our cohort. Both ablation methods were associated with a significant improvement in the EHRA and NHYA stage, which is probably not related to the energy source used but the result of the freedom from atrial fibrillation. In terms of success both techniques seem to be comparable, therefore, the decision which technique should be utilized should depend on the expected arrhythmic substrate.

### Safety

Unlike other ablation methods, PFA seems to have a high specificity to cardiomyocytes [6]. In the MANIFEST-PF Registry and in the recently presented MANIFEST-17 K study, the largest PFA study so far with more than 17,000 patients, no cases of pulmonary vein stenosis, esophageal ulcerations, or atrio-esophageal fistula were observed, and only one patient (0.6%) did suffer from a persistent phrenic palsy, that partially resolved 1 year after PVI [8] [19]. On the other hand, vHPSD-RF creates shallower RF lesions and may thereby contribute to better safety and less damage to neighboring organs, especially the esophagus and phrenic nerve [20]. In agreement with earlier research, both ablation methods demonstrated a good safety profile. Aside from one case of stroke that occurred in the vHPSD-RF group due to the early interruption of the oral anticoagulation 2 days after the ablation against our recommendations, no further major complications were observed. However, it is worth mentioning that detecting differences regarding complications with very low incidence, such as atrio-esophageal fistula, requires studies with larger number of patients [21].

### Limitations

First, our findings are inherently limited by the retrospective, non-randomized study design, the relatively small number of patients, and the short follow-up period. Second, most patient did not receive a continuous or standardized rhythm monitoring between discharge and the 6-month follow-up visit. Additionally, a total of 16 patients (19,5%) did not attend their scheduled follow-up visits. To evaluate the long-term efficacy of these novel methodologies, the acquisition of standardized longer-term follow-up data is imperative and remains pending.

### Conclusions and future research

PFA and vHPSD-RF emerge as novel promising ablation methods in the management of AF. While both methods facilitate fast and safe pulmonary vein isolation, PFA stands out for its significantly shorter procedure and ablation times. Given its speed, selectivity, and feasibility, PFA may evolve into the preferred ablation method for index PVI in the near future, especially for patients with high sedation risk, where short procedural duration is crucial. The large catheter footprint of the PFA system used in this study (Farapulse™) restricts its application to the isolation of pulmonary veins. Conversely, vHPSD-RF using the single-tip QDOT micro-catheter (Biosense Webster, USA) is capable of addressing ablation targets beyond the pulmonary veins and is associated with lower radiation exposure. Both methods exhibited very good safety and short-term effectiveness profiles in this retrospective single-center study; however, larger prospective randomized clinical trials with longer follow-up periods are needed to further validate our findings.

## Data Availability

The data that support the findings of this study are not openly available due to reasons of patients privacy and are available from the corresponding author upon reasonable request.

## Abbreviations

PVI: Pulmonary vein ablation
AF: Atrial fibrillation
RF: Radiofrequency
PFA: Pulsed-field ablation
vHPSD-RF: Very high-power short-duration radiofrequency
CRA: Cryoballoon ablation
LA: Left atrium
DAP: Dose area product
TIA: Transient ischemic attack
TEE: Transesophageal echocardiography
etCO2: End-tidal carbon dioxide
TSP: Transseptal puncture
PE: Pericardial effusion
ACT: Activated clotting time
CTI: Cavotricuspid isthmus
EHRA: European Heart Association
NYHA: New York Heart Association
LAVI: Left atrial volume index
AI: Ablation index
